# Progress Toward a Gonococcal Vaccine: The Way Forward

**DOI:** 10.3389/fimmu.2019.02417

**Published:** 2019-10-15

**Authors:** Michael W. Russell, Ann E. Jerse, Scott D. Gray-Owen

**Affiliations:** ^1^Department of Microbiology and Immunology, University at Buffalo, Buffalo, NY, United States; ^2^Department of Microbiology and Immunology, F. Edward Herbert School of Medicine, Uniformed Services University, Bethesda, MD, United States; ^3^Department of Molecular Genetics, University of Toronto, Toronto, ON, Canada

**Keywords:** *Neisseria gonorrhoeae*, immunity, vaccine, antigens, antibodies, T cells, cytokines

## Abstract

The concept of immunizing against gonorrhea has received renewed interest because of the recent emergence of strains of *Neisseria gonorrhoeae* that are resistant to most currently available antibiotics, an occurrence that threatens to render gonorrhea untreatable. However, despite efforts over many decades, no vaccine has yet been successfully developed for human use, leading to pessimism over whether this goal was actually attainable. Several factors have contributed to this situation, including extensive variation of the expression and specificity of many of the gonococcal surface antigens, and the ability of *N. gonorrhoeae* to resist destruction by complement and other innate immune defense mechanisms. The natural host restriction of *N. gonorrhoeae* for humans, coupled with the absence of any definable state of immunity arising from an episode of gonorrhea, have also complicated efforts to study gonococcal pathogenesis and the host's immune responses. However, recent findings have elucidated how the gonococcus exploits and manipulates the host's immune system for its own benefit, utilizing human-specific receptors for attachment to and invasion of tissues, and subverting adaptive immune responses that might otherwise be capable of eliminating it. While no single experimental model is capable of providing all the answers, experiments utilizing human cells and tissues *in vitro*, various *in vivo* animal models, including genetically modified strains of mice, and both experimental and observational human clinical studies, have combined to yield important new insight into the immuno-pathogenesis of gonococcal infection. In turn, these have now led to novel approaches for the development of a gonococcal vaccine. Ongoing investigations utilizing all available tools are now poised to make the development of an effective human vaccine against gonorrhea an achievable goal within a foreseeable time-frame.

## Introduction

The concept of vaccinating against gonorrhea dates back to the earliest years following the discovery of *Neisseria gonorrhoeae* as the causative agent of this sexually transmitted disease in the late nineteenth century. In the early twentieth century, numerous attempts were made to treat gonorrhea by injecting various whole cell vaccines in the belief that these would promote opsonophagocytosis (1), which had been just been discovered by Wright and Douglas (2). However, none of these approaches were successful, and most were poorly controlled and inadequately described efforts. The advent of chemotherapy, first with sulfonamides (1936) and soon followed by penicillin (1943), afforded dramatically successful treatment, obviating the need for vaccine development. However, resistance to these antibiotics soon emerged, a pattern repeated as each subsequent antibiotic was introduced, until the present when current U.S. guidelines call for dual treatment with a cephalosporin plus azithromycin (3). Unfortunately, resistance to both of these drugs is now emerging and several instances of treatment failure to such combinations have now been reported (4). As a result, authorities such as the U.S. Centers for Disease Control and Prevention and the World Health Organization have called for renewed efforts at gonococcal vaccine development.

An outside observer might reasonably ask why we do not already have a vaccine against gonorrhea, given that *N. gonorrhoeae* has been known as the causative agent for such a long time. However, a combination of three major factors has contributed to this situation. First, like a number of other infections, an episode of gonorrhea does not confer protective immunity against repeat infection, which is a relatively common occurrence. Consequently, in the absence of a clear state of immunity to gonorrhea in humans, it has not been possible to define the determinants or even correlates of immunity. Secondly, gonorrhea is a uniquely human disease, and *N. gonorrhoeae* has no known natural hosts other than humans. Thus, despite various efforts, it has been difficult to establish an animal model of infection, especially one that would replicate the human disease, in which immune responses and vaccines can be evaluated. As will be discussed below, this situation has been rectified, at least in part, by the development of a female mouse model (5), which has now been used in several laboratories to reveal unexpected aspects of immunity to *N. gonorrhoeae*. Finally, *N. gonorrhoeae* has long been known to display highly variable antigenicity, such that most of its main surface antigens continually evolve their sequence and/or reversibly phase-vary their expression on and off. While many pathogens utilize antigenic variation as a strategy of immune evasion, few do so to the extent that *N. gonorrhoeae* displays. As outlined below, elegant studies over the past three decades have revealed several mechanisms that independently promote this antigenic variation. This genetic plasticity complicates the assessment of specific immune responses to infection since even the same isolate will be antigenically different upon repeated passage. In addition, many gonococcal antigens are similar to those found in other neisserial species, including the closely related human pathogen *Neisseria meningitidis* and a variety of commensal *Neisseria* species commonly found in the human mouth and pharynx. Thus, most adults display serum anti-gonococcal antibodies regardless of whether or not they have been exposed to *N. gonorrhoeae*. As a result, it has proven impossible to define serological criteria—antibody levels—that unequivocally indicate previous exposure to *N. gonorrhoeae*, or that might indicate protective immunity.

## Phase and Antigenic Variation

Any consideration of gonococcal infection must take into account the unrivaled variability of this organism. *N. gonorrhoeae* is well-known for its extraordinary capacity to vary its surface antigen composition, both between strains and within the same strain over time. In the case of gonococcal type IV pili, promoterless copies of the genes that encode the major pilus subunit provide a silent repository of sequences that can be shuffled into an expression locus to generate new antigen variants. Aside from this intra-chromosomal recombination, *N. gonorrhoeae* is also highly competent for genetic transformation, allowing it to sample DNA fragments present in the mucosal environment in which it exists. This is facilitated by two different processes: a type IV secretion system that actively pumps single-stranded DNA into the environment (6) and a DNA uptake system that effectively binds and takes up DNA so that it may recombine into the chromosome if homologous sequences are present. This latter process is particularly efficient with DNA fragments containing short (~10 base pair) non-palindromic conserved “uptake sequences” widespread throughout the neisserial genome (7, 8), which allow the bacteria to preferentially select DNA originating from pathogenic or commensal *Neisseria* species. This allows the rapid sharing of new genetic features, such as new resistance alleles when under antibiotic selection or variant alleles under adaptive immune pressure, and makes the genetic structure of the *N. gonorrhoeae* population panmictic rather than clonal.

Augmenting the effect of recombination-dependent antigen variation, the expression of many proteins is reversibly switched off and on through a process known as phase variation (9). The ongoing phase and antigenic variation, occurring independently in each bacterial cell, has the effect of continuously diversifying the population with respect to the surface epitopes that they express and, in many cases, the phenotype of the bacteria. It is, therefore, a combination of immune-based selection and the fitness conferred by each combination of attributes expressed that determines which variants dominate the population. The genetic processes that mediate neisserial genetic plasticity are the focus of an excellent recent review (10). Herein, we will briefly consider several different surface antigens that have been considered as vaccine targets to illustrate how each of these processes are accomplished, how they contribute to infection and immune evasion, and why they must be considered in vaccine development.

### Porin

The porin, PorB, is the most abundant outer-membrane protein and is constitutively expressed by *N. gonorrhoeae*. Each isolate possesses a single *porB* allele, which encodes an outer membrane-expressed β-barrel with 16 membrane-spanning sequences and 8 surface-expressed loops (11). Targeting PorB in a vaccine is confounded by extensive variation within the sequence and length of its surface-exposed loops expressed by different strains, which provides the basis for gonococcal serogroup and serovar typing schemes (12). Aside from their antigenic differences, *porB* alleles can differentially contribute to processes including serum complement resistance (13, 14), apoptosis (15–17), and host cellular invasion (18, 19). In addition, properly folded PorB, as found in its native outer membrane environment, has a potential role in suppressing the adaptive immune response by inhibiting dendritic cell stimulation of CD4+ T cell proliferation (20). While the *porB* allele expressed is considered to be a relatively stable phenotype within a strain, genetic transformation and recombination between co-infecting strains generates a PorB mosaicism that results in variants with new antigenic and, presumably, functional characteristics (21). This poses a challenge with regards to targeting the antigen; however, PorB has a potent TLR2-dependent adjuvant effect that stems from its tendency to form self-aggregating micellular structures, suggesting that it may be an interesting vaccine component (22).

### LOS Glycan Structure

While phase variation most obviously alters protein expression, the differential expression of enzymes can also alter glycan epitopes on the gonococcal surface. The glycan side-chains of the lipo-oligosaccharide (LOS) are determined by cytosolic glycosyltransferases, which are subject to phase-variable expression (23, 24). This process occurs through a slip-strand mispairing of homopolymeric nucleotide tracts within the regions encoding LOS glycosyltransferase genes, which leads to addition or subtraction of the repeated oligonucleotide sequences and, thereby, expression or not of the enzyme based upon whether its coding sequence is in or out of the translation reading frame (23, 25–27). Aside from influencing immune cross-reactivity between isolates, LOS variation can also directly impact gonococcal association with epithelial cells (28) and the recruitment of serum complement regulatory proteins that protect against the bactericidal activity of blood (29, 30). A monoclonal antibody, 2C7, generated from a mouse immunized with gonococcal outer membranes, recognizes a lactose residue substituted on heptose I or heptose II of gonococcal LOS (31). This antibody also reacts with 95% of fresh clinical isolates (31), and it enhances clearance of *N. gonorrhoeae* infection in a mouse lower genital tract infection model (32), suggesting that such phase-variable antigens might be effective vaccine targets if there is sufficient selection for their expression during natural infection. However, the 2C7 epitope can be lost due to phase-variable expression of LOS glycosyltransferases (33).

### Opa Proteins

The gonococcal colony opacity-associated (Opa) proteins are a large family of integral outer membrane proteins each consisting of a β-barrel with 8 highly conserved transmembrane sequences that display four surface-exposed variable loops (34). A single gonococcal isolate possesses ~11 different *opa* genes, the expression of each being independently and stochastically regulated by phase variation such that the bacteria may express no Opa proteins or any combination of one or more Opa variants. This on-off switching occurs because, although each locus is constitutively transcribed at a high level, the protein-coding sequence can “slip” in or out of the translational reading frame due to the addition or loss of one or more pentanucleotide (CTCTT) repeat sequences in the structural gene that encodes the amino-terminal leader peptide (35). As with other repeat sequences that lead to phase-variable gene expression, the addition or subtraction of pentanucleotide repeats can occur during chromosomal replication at cell division or by homologous recombination between *opa* loci, both of which contribute to frequent (10^−3^ per generation) changes in Opa phenotype. The large number of *opa* genes also allows ongoing intra-chromosomal and horizontal (inter-strain) recombination between the conserved membrane-spanning or periplasmic sequences, resulting in the replacement of one or more surface loops (36); this explains the hundreds of *opa* alleles described to date. Remarkably in the context of this variability, most Opa variants retain protein-protein binding to human carcinoembryonic antigen-related cellular adhesion molecules (CEACAMs), their human cellular receptors (37–43), and retain their lectin-like function that mediates inter-bacterial aggregation (44). This conservation of function allows persistent expression of these binding phenotypes while allowing the bacteria to evade Opa variant-specific immunity.

### Pilus

The type IV pili are, perhaps, the most remarkable structures on the gonococcal surface, extending ~1 μm from the outer membrane to allow human cellular attachment before retracting to allow tight apposition of the bacterial and host cell surfaces. The pilus fiber is composed of a helical array of pilin proteins, the preponderance being the major pilin protein, PilE (45, 46). Aside from the full length *pilE* expression locus, there are ~20 transcriptionally “silent” *pilS* loci (47, 48). These are effectively repositories of variant pilin sequences because each encodes the surface-exposed variable regions interspersed by conserved sequences that make up the pilin protein core, but have no promoter sequence or start codon. Rather than being mediated by simple homologous recombination, this process involves a “gene conversion” event that allows alteration of the target expression locus while also retaining the variable sequences within the “donor” *pilS* locus (49). This ongoing high-frequency variation assures that the bacteria display an ever-changing array of epitopes that makes it impossible for the adaptive immune response to catch up to.

The remarkable ability of *N. gonorrhoeae* to generate antigenic variation while retaining functionality of its surface proteins has engendered the belief that, while an antibody response might be induced by infection, this would be rendered ineffective as subpopulations of bacteria are always present that can avoid antibody recognition. This, along with more recent findings concerning gonococcal immune evasion, has led to fears that it might not be possible to elicit immune protection. However, as discussed below, evidence is now accumulating that, while *N. gonorrhoeae* possesses the ability to subvert the development of an effective immune response, an appropriately targeted *N. gonorrhoeae*-specific immune response can be protective.

## Previous Vaccine Efforts

Early work with chimpanzees provided the first indication that gonococcus-specific immunity can be achieved. Experimentally inoculated male chimpanzees were observed to clear urethral infection within several weeks, and a 3-log higher infectious dose was required to re-infect the animals 1 week after the infection had cleared. These animals were not immune when re-exposed 2 years after the primary infection, suggesting that this immunity waned over time (50). Subsequent studies with a killed whole-cell gonococcal vaccine reduced susceptibility to gonococcal infection, as indicated by the requirement for an increased infectious dose (51), suggesting that parenteral immunization might be a reasonable approach.

Only two vaccine candidates have been tested in field trials with humans. The first was a parenterally delivered heat-killed, partially lysed whole-cell vaccine that induced serum antibody titers in 90% of subjects but showed no protection based upon a similar number of individuals infected within 12 months after vaccination (52, 53). The second gonorrhea vaccine tested in humans was a purified pilin vaccine that was delivered intradermally using a two-dose regimen separated by 2 weeks to over 3,000 US military personnel in Korea. No difference was found between groups given the vaccine vs. a placebo, with 108 and 102 subjects, respectively, acquiring gonorrhea 2 weeks after inoculation (54). The likelihood that antigenic variability of gonococcal pili was responsible for, or at least contributed to, the failed study is supported by reports that this vaccine showed efficacy during in-house trials using the homologous strain (55) but not a heterologous strain (56). Notably, pilus-specific mucosal antibodies that blocked gonococcal adherence to human cells appeared to be a correlate of protection in this trial (57), providing perhaps the clearest indication of how immunity might be achieved. Regardless, the point that efficacy was observed in the homologous challenge trial should not be ignored since it counters the often-held assumption that immunity to *N. gonorrhoeae* was not achievable in humans.

The momentum toward developing a gonococcal vaccine was lost with onset of the HIV epidemic in the early 1980s, which diverted investigators and research funding away from gonorrhea and other STDs. The focus on HIV also meant that chimpanzees were no longer available for gonococcal vaccine studies, and there was not yet a small animal model for systematic testing of candidate vaccines and the immune responses they elicit. These events, when combined with the failure of the first human trials, had the unfortunate effect of reducing the interest of the pharmaceutical industry and other agencies, and of discouraging further gonococcal vaccine research for decades. With recent increases in the global burden of gonococcal infection and the emergence of multidrug resistant strains, this situation has now begun to turn around.

## Natural Infection Does Not Induce Protective Immunity: Studies With Humans

Studies in humans have been complicated by numerous factors including: logistical difficulties in recruiting volunteers willing to adhere to study protocols; practical considerations of defining matched infected and control groups to account for confounding factors; the frequent occurrence of “asymptomatic” infections in both females and males, which creates uncertainty over the time elapsed between exposure and diagnosis of infection; different states of infection in males and females, ranging from uncomplicated urethritis or cervicitis to complicated upper tract infection (prostatitis or epididymitis in males, endometritis, and salpingitis with pelvic inflammatory disease in females) and, more rarely, disseminated systemic infection; the frequent occurrence of co-infections; the unreliability of subjects' self-reporting previous infection, which is further confounded by asymptomatic infection; and the availability of funds. Ethical considerations also impose constraints because subjects presenting at clinics must obviously be offered treatment upon diagnosis, thereby limiting longitudinal studies aimed at understanding the natural progression of infection and immunity. Experimental studies involving challenge infections are limited to males because of the risk of complications and sequelae in females. However, even in males, the requirement to treat the infection shortly after symptoms develop means that only short-term studies focused on the establishment of infection are realistically possible.

Despite the challenges associated with studying infection in humans, numerous studies have attempted to comprehend the interaction of *N. gonorrhoeae* with various components of the human immune system. From these, certain conclusions can be drawn, understanding of course that the relative contribution of these effects during natural gonococcal infection remains poorly understood.

Antibody and cell-mediated (cytokine) responses to documented, uncomplicated gonococcal infection in both women and men are weak and of short duration (58, 59). Assays of antibody and T cell responses are complicated by the extensive antigenic variability of *N. gonorrhoeae*, which means that it is difficult to define a standard antigen preparation against which to evaluate responses. However, even when responses were evaluated against homologous gonococcal isolates, it became clear that antigenic variation does not explain the absence of response since reactivity with the homologous strain was little more than that measured against a prototypical laboratory-adapted strain (58). These observations, coupled with the finding that responses tended to dissipate after a few weeks, and were apparently no greater in individuals documented to have had previous infections, led to the proposition that *N. gonorrhoeae* was somehow capable of interfering with the normal course of an adaptive immune response (59). In contrast, men experimentally infected with *N. gonorrhoeae* in the urethra were found to show elevated levels of inflammatory cytokines (IL-8, IL-6, IL-1β, and TNF) in plasma and urine (60). In a subsequent study on experimentally infected men, increased titers of anti-LOS IgG antibodies were observed in some subjects, but no evidence was seen for enhanced resistance to re-infection with the same gonococcal strain 2 weeks after treatment of the initial infection, compared to previously uninfected controls (61).

The lack of specific protective immunity stands in marked contrast to the strong inflammatory response elicited during symptomatic infection, which is evident by the massive influx of neutrophils, as well as by the inflammatory cytokine responses noted above. However, insufficient attention has been given to the different states of infection in men and women, and the possibility that host adaptive responses might accordingly be different. Indeed, the recognition that women who suffer from severe cases of PID have heightened gonococcal-specific antibodies and antibody-dependent serum bactericidal activity (62), as well as protection against salpingitis (63), clearly demonstrates that context is important. Given that systemic gonococcal infection can also elicit a memory immune response (64), it seems reasonable to consider that an adaptive response might depend on gonococcal tissue penetration during these more severe manifestations of infection. This requirement would explain why most gonococcal lower urogenital tract, pharyngeal, or rectal infections, which are either asymptomatic or induce little tissue damage, do not elicit a memory response. Nevertheless, when considered together, these findings collectively support the hypothesis that *N. gonorrhoeae* can interfere with the immune response to uncomplicated infection at the mucosal surfaces.

### Humoral Immunity

Whether gonococcus-specific antibodies are present or not, *N. gonorrhoeae* has been shown to be capable of obviating complement-dependent bacteriolysis, and several mechanisms have been described. Gonococcal sialylation of its LOS by means of its surface-expressed sialyl-transferase (65), which utilizes host-derived cytidine monophosphoryl-*N*-acetyl-neuraminic acid (CMP-NANA) as substrate, inhibits complement (C3) deposition to facilitate gonococcal survival in human blood (66, 67) and in mice (68, 69). Sialylation of LOS also enhances binding of human complement regulatory factor H to PorB (70). Some naturally occurring variants of gonococcal PorB can interfere with the alternative and classical pathways of complement activation by binding to factor H or C4b-binding protein (C4BP), respectively (71, 72). It is important to note that PorB binding is specific for human C4BP and factor H, so these effects may not be evident in serum bactericidal assays if non-human complement is used or in most animal infection models. Gonococcal NspA (neisserial surface protein A) also binds human factor H (73), but the gonococcal homolog of the meningococcal factor H-binding protein lacks a signal peptide and therefore is not available on the cell surface to bind factor H (74).

While the mechanism remains poorly understood, the highly immunogenic reduction-modifiable protein (Rmp), which sits closely associated with porin on the gonococcal cell surface, elicits antibodies that block complement activation by IgG antibodies against LOS or porin (75). This mechanism, which serves to enhance gonococcal survival in mice as well as in human serum (76), has led to the hypothesis that protective immunity can be defined by the ratio of antibodies to LOS and porin over antibodies to Rmp (76). However, in the absence of a state of immunity to gonococcal infection in humans, this hypothesis has not been confirmed or refuted.

Finally, based upon parallels with studies done with *N. meningitidis*, it appears likely that the gonococcal IgA1 protease also confers a protective effect. Specifically, IgA antibodies specific for the meningococcal capsular polysaccharide can block complement-mediated bacteriolysis caused by IgM or IgG antibodies to the same antigen (77). In this case, the Fabα fragments generated upon cleavage of IgA1 antibodies by the meningococcal IgA1 protease mediate this effect through their ability to mask epitopes and, thereby, prevent binding of functional full length (Fc-containing) antibodies (78, 79). As all strains of *N. gonorrhoeae* constitutively produce a homologous IgA1 protease (80), it seems likely that a similar mechanism operates with gonococcal infection, but evidence in support of this has not been forthcoming (81). Additionally, it has been proposed that gonococcal IgA1 protease enhances intracellular survival by cleaving the lysosomal protein, LAMP1 (82, 83), suggesting that this virulence factor has multiple infection-enhancing effects.

### Interactions With Phagocytes

The hallmark of gonorrhea has long been the presence of a purulent discharge that consists primarily of neutrophils, many of which are associated with apparently intact diplococci. It is well-known that phagocytic uptake and killing of bacteria is greatly enhanced when the bacteria are coated with opsonins consisting of antibodies and/or complement. The “professional” phagocytes, i.e., neutrophils and macrophages, express receptors for the Fc regions of IgG (FcγRs) and IgA (FcαRI), which carry signaling immuno-tyrosine activation motifs (ITAMs) on their cytoplasmic domains, and for C3b. These receptors not only promote attachment of bacteria to the phagocytes, but the Fc receptors also stimulate intracellular signaling events that result in release of bactericidal granules into the phagocytic vesicles and trigger the oxidative burst that generates reactive oxygen species, thereby leading to killing of ingested bacteria. However, the interaction of *N. gonorrhoeae* with human neutrophils is not necessarily dependent on opsonization, since it can also be mediated by gonococcal pili and Opa protein adhesins binding to their host cellular receptors, especially during the early stages of infection. Gonococcal variants lacking these adhesins are less readily ingested and killed by neutrophils (84, 85). In later stages when inflammation has been induced with exudation of plasma proteins, the increased availability of complement components, especially C3, allows the binding of C3b to the gonococcal surface by alternative pathway activation (86) or even direct proteolytic cleavage of C3, and subsequent binding to C3b receptors on neutrophils.

The adhesin-mediated interaction with phagocytes is host-restricted, since non-opsonized gonococci avoid capture by mouse neutrophils regardless of whether pilus or Opa proteins are expressed. However, gonococci are effectively engulfed if transgenic mouse neutrophils express human CEACAMs that function as the cellular receptors for the neisserial Opa proteins (87, 88). Particularly notable in this regard, CEACAM3 is an innate decoy receptor expressed exclusively on human neutrophils. The CEACAM3 extracellular domain mimics epithelial cell-expressed CEACAMs, allowing effective gonococcal binding. Because its cytoplasmic domain carries a signaling ITAM motif similar to those on Fc receptors, Opa protein-dependent engagement of CEACAM3 triggers bacterial phagocytosis (89–93), neutrophil degranulation and oxidative burst (40, 90, 91), as well as a pro-inflammatory chemokine response that drives ongoing neutrophil recruitment to the infected tissues (87, 88, 94). Depending upon the bacterial burden and presumably other factors, this CEACAM3-dependent response can either effectively clear the infection or contribute to the self-propagating inflammation that typifies gonococcal disease. However, the phase-variable expression of Opa proteins, some of which do not recognize CEACAMs, always maintains some gonococci that evade capture by neutrophils (43, 95, 96) and, thereby, avoid this bactericidal response (43, 97–99). These opposing effects drive *in vivo* selection for Opa variants that do not engage CEACAM3 but still allow epithelial cell attachment (42).

Opsonin-dependent uptake of gonococci by phagocytes is limited by the paucity of IgG (and IgA) antibodies, the production of which is suppressed by *N. gonorrhoeae* (as discussed below) and rendered ineffective by antigenic variation, and by several gonococcal mechanisms that inhibit complement activation or deposition. For example, sialylation of LOS by means of host-derived CMP-NANA inhibits C3b deposition on the gonococcal surface, thereby diminishing phagocytic uptake (68, 100, 101). While the gonococci are usually sialylated during infection, most *in vitro* phagocytosis experiments have not utilized *N. gonorrhoeae* grown in the presence of CMP-NANA to ensure that it is sialylated, which may result in misleading conclusions. In addition, *N. gonorrhoeae* resists intracellular phagocytic killing in several ways, including suppression of the oxidative burst, resistance to non-oxidative killing mechanisms, and the production of lytic transglycosylases (102–106). The extent to which opsonization of *N. gonorrhoeae* by specific IgG (or IgA) antibodies and/or complement can overcome its ability to avoid intracellular killing requires further investigation.

While largely overlooked due to the striking role that neutrophils play in gonorrhea, recent work has begun to illuminate the role that other phagocytes play during infection. Gonococcal infection of primary macrophages promotes their survival and stimulates their expression of pro-inflammatory cytokines (107), suggesting that these innate cells may play an important role in the recruitment of neutrophils during the early stages of infection. However, exposure to *N. gonorrhoeae* promotes human monocyte differentiation into macrophages with a tissue repair (M2) phenotype that are unable to stimulate T cell proliferative responses (108, 109). The relative contributions of inflammatory and inhibitory macrophage responses might influence the intensity of the overall response and thereby affect the outcome of gonococcal infection.

A mechanistic explanation for the suppressive effect of *N. gonorrhoeae* on macrophages remains to be determined. However, it at least functionally resembles that seen with human monocyte-derived dendritic cells, where Opa protein-dependent binding to the immune co-inhibitory receptor CEACAM1 suppresses their normal maturation into antigen-presenting cells (110). This, when coupled with the fact that Opa binding to CEACAM1 on CD4+ T cells effectively inhibits their ability to be stimulated by T cell receptor engagement or cytokine exposure (111, 112), provides a clear indication that gonococci have the capacity to directly interfere with the adaptive response in humans. However, rather than simply suppressing immunity, gonococci also appear to misguide the response by virtue of their ability to drive a robust T cell-independent activation of the IgD- and CD27-expressing subset of innate-like human B cells, which results in the rapid accumulation of broadly-reactive (not *Neisseria*-specific) low affinity IgM antibodies (113). It is enticing to consider that this effect might promote a localized increase in irrelevant antibodies during human genital infection with *N. gonorrhoeae*, an effect that would both be ineffective and have a suppressive effect on other B cells.

## Animal Models

The classic paradigm, that vaccination seeks to mimic a natural infection to induce immunity without causing disease, is clearly inapplicable to gonorrhea because the essential premise, that natural infection induces immunity, is not true. Instead, it becomes necessary to comprehend how a reactogenic inflammatory infection such as gonorrhea interacts with the immune system so as to avoid inducing an effective response. An effective vaccine will need to avoid this same outcome. However, experimental investigation of gonococcal pathogenesis and the host response, and understanding of the factors that affect these responses, have been severely limited by strict human specificity of *N. gonorrhoeae*, which challenges animal modeling of this infection. Ethical considerations prohibit the study of the human disease in the absence of treatment, and various efforts to infect other animal species in a manner resembling the human disease have been unsuccessful (114). A possible exception is the reported genital infection of a male chimpanzee and the subsequent sexual transmission of infection to a female (115), but experimenting on this species would be prohibitively expensive and, in any case, is now impermissible. Without a protection model, vaccine development has been stunted because efficacy testing has not been possible. Fortunately, this situation has been partially rectified by the development of the estradiol-treated female mouse model of genital tract gonococcal infection (5), which is robust, genetically tractable, and has been extensively used in various laboratories over the past two decades.

### Inbred Mouse Strains

In the 1980s it was reported that female mice can be transiently colonized with *N. gonorrhoeae* if inoculated in the proestrus stage of the estrous cycle, when vaginal neutrophils and commensal flora are low (116–118). Colonization cleared, however, upon transition to the late estrus/metestrus stages. These observations led to the demonstration that germ-free BALB/c mice treated with 17-β-estradiol could be vaginally colonized with *N. gonorrhoeae* for several weeks, whereas mice that were instead treated with progesterone resisted infection (119). This model was made more broadly practical by subsequent work demonstrating that conventional (not germ-free) 17-β-estradiol-treated BALB/c mice could be infected if estrogen-induced overgrowth of their vaginal microbiota were suppressed by antibiotic treatment (5). It seems likely that the high numbers of neutrophils present in the vaginal lumen during the progesterone-dominant diestrus stage explain the resistance of progesterone-treated mice to gonococcal infection, while the bloom in commensal microbes that normally occurs due to increased glycogen secretion by vaginal epithelium during estrus demands the use of antibiotics in estradiol-treated mice. However, given that the sex hormones can influence immune cell function (120, 121), as well as epithelial expression of innate inflammatory mediators (122, 123) and genital tissue architecture (124), other factors undoubtedly contribute to the success of this approach. Nonetheless, the impact of female hormonal cycling on gonococcal infection in mice (125) reflects clinical observations that gonococcal abundance also appears to increase during the proliferative (high estrogen) stage and then wane during the luteal (high progesterone) stage of the menstrual cycle in women (126, 127). Likewise, there are broad similarities in vaginal carbon (lactate and glucose) availability (128), oxygen tension, physicochemical properties of mucus, and interactions with commensal microbiota (129–131). However, pH differs: mouse vaginal pH ~6.2, human vaginal pH ~4.0, although human cervical pH ~6.0 (130). Iron restriction differs in that *N. gonorrhoeae* can extract iron from human transferrin and (in some strains) lactoferrin, whereas it is unable to utilize murine transferrin or lactoferrin (132). Nevertheless, the mouse infection model affords a valuable surrogate for evaluating both innate and adaptive immune responses that cannot be adequately modeled *in vitro*, and is currently the only available model in which the interaction of *N. gonorrhoeae* and an intact mammalian immune system can be studied. Table 1 summarizes the similarities and differences between gonococcal infection in humans and mice.

**Table 1:** Similarities and differences in gonococcal infection between mice^*^ and humans.

**Aspect**	**Mouse**	**Human**
Antibody response	None	Weak to none
Th1 response	No	No
Th2 response	No	No
Th17 response	Yes	Some evidence
Memory development	No	No
Protection against reinfection	No	No
Neutrophil influx	Depends on strain	Typical in symptomatic infection
Duration of infection	~1–3 weeks	Several months?
Primary site of infection	Vagina and cervix	Cervix
Impact of reproductive hormones	Cyclical fluctuations in recovery of gonococci	Gonococcal recovery zero or low in luteal phase, increased in proliferative phase
Disease	Inapparent or inflammatory cervico-vaginal infection, depending on mouse strain. Ascending infection in ~20% of mice following vaginal inoculation; endometrial infection in most mice following transcervical inoculation	Asymptomatic (m, f); Cervicitis (f), urethritis (m); Endometritis, salpingitis (f); Pelvic inflammatory disease (f); Prostatitis, epididymitis (m); Disseminated (m, f)

*f, female; m, male*.

* *Male mouse model not available*.

While the BALB/c infection model is the most well-established, certain other (but not all) mouse strains can be similarly infected (5, 133). Notable in this regard is that C57BL/6 and BALB/c mice are similarly permissive to infection, but the former do not reveal the neutrophil response shown by the latter (133), perhaps suggesting that a marked neutrophil response does not greatly influence the course of infection in mice. While it is also tempting to contemplate that the relative susceptibility of each inbred strain is determined by varying innate or adaptive immune responses stemming from their genetic differences, it is also possible that susceptibility is related to differences in the genital microbiota carried by the mice, which in part also varies with their source. This is particularly interesting considering that *Lactobacillus*-derived lactate is a preferred carbon source for *N. gonorrhoeae* (128), that components of the microbiota can influence gonococcal growth *in vitro* (129, 134), and that microbe-derived sialidases can modify the gonococcal LOS to facilitate its association with epithelial cells (135).

### Genetic Manipulation of Mice

Aside from the obvious benefit of being able to experimentally infect mice with naturally occurring or recombinant gonococcal strains, the murine system also allows genetic manipulation of the host. This has been particularly useful for efforts to understand determinants of immunity. For example, isogenic mouse lines lacking the endotoxin receptor, Toll-like receptor 4 (TLR4), have a substantially higher burden of *N. gonorrhoeae* than do wild-type controls after vaginal infection, and this correlates with an exaggerated classical (TNF, CXCL1/KC, and CXCL2/MIP-2) inflammatory response but a diminished IL-17 response in TLR4-deficient mice (136–138). While it might be expected that innate recognition of microbe-associated molecular pattern (MAMP) molecules such as LOS would promote immune defense, these findings provide important mechanistic context for an emerging recognition that Th17-driven innate responses to gonococcal infection occur concomitantly with suppression of Th1/Th2-driven adaptive responses (138–140), which is discussed below. This disconnect between innate and adaptive immunity is fundamental to understanding why *N. gonorrhoeae* is such a successful human parasite, and exemplifies how mouse models provide important insight that could not emerge from other experimental approaches.

When modeling any infection, whether using *in vitro* cellular infection or a non-natural host, it is imperative that any results be considered in the context in which they are being observed. Mouse infection models provide an opportunity to consider infection within a physiologically relevant mucosal environment, including a fully functioning immune system, conditions which are impossible to replicate outside of an animal. However, the fact that many gonococcal virulence factors only bind to their human-derived targets means that their contribution to infection cannot be appreciated in a wild type mouse. Given that the first step in infection is anchorage to host tissues, the fact that gonococcal pilus and Opa protein adhesins do not bind mouse tissues provides the most obvious example of this point. The elegant means by which *N. gonorrhoeae* overcomes nutritional immunity, including both iron (141, 142) and zinc (143) acquisition systems, and by which it binds to human proteins to evade serum complement-dependent killing (144, 145), are rendered ineffective in the mouse.

Fortunately, the genetic tractability of mice has allowed direct investigation of the impact that well-characterized gonococcal effector proteins play *in vivo*. This strategy has recently been applied for the generation of transgenic mice expressing different combinations of human CEACAMs, which are the receptors for most neisserial Opa protein adhesins. While an enormous amount of primary and immortalized human cell-based work has described how individual human CEACAMs can each contribute to the outcome of gonococcal interactions with different cell types (37–41, 89–91, 93, 99, 110, 111, 146–154), the gonococcal Opa proteins do not bind mouse CEACAM1 and mice do not express CEACAM3, CEACAM5, or CEACAM6, so all four targets are effectively absent. Transgenic mice have now been generated to encode various combinations of these receptors, and each CEACAM appears to be expressed with a cellular distribution reflecting that in humans, including CEACAM1, CEACAM3, and CEACAM6 on neutrophils, CEACAM5 on vaginal epithelium, and CEACAM1 on the endometrium (88, 94). Consistent with *in vitro* cell-based studies, human CEACAM5 expression promotes an intimate Opa protein-dependent attachment to mucosal tissues and an extended duration of colonization within the female lower genital tract (94). In addition to the direct effect of Opa binding on epithelial attachment, this outcome is facilitated by decreased exfoliation of infected epithelial cells due to the CEACAM-dependent upregulation of β1 integrin-mediated binding to their underlying extracellular matrix (155). However, the effect of CEACAM expression depends on the exposed tissue and stage of the female reproductive cycle since, for example, uterine inflammation occurs in transcervically infected wild-type mice in diestrus (156) but occurs only in the presence of human CEACAM1 when the mice are in estrus (94, 157). Moreover, the integration of CEACAM-dependent effects determines the outcome of infection since neutrophil expression of CEACAM3 promotes inflammation and phagocytic clearance of the gonococci, effectively opposing the infection-promoting contribution of epithelial CEACAMs (88, 94). What was not foreseen by *in vitro* studies was that the neutrophil response to CEACAM3 binding includes their triggering of a potent pro-inflammatory transcriptional program that stimulates an ongoing recruitment of neutrophils, an effect that may help drive the clinical manifestations of gonorrhea (43, 87, 88). Ongoing efforts to generate mice co-expressing human CEACAMs along with other factors targeted by neisserial virulence factors, including the human iron-sequestering proteins transferrin (141) and lactoferrin (142), zinc-sequestering calprotectin (143), and the complement regulatory proteins factor H (144) and C4 binding protein (145), will allow further appreciation of how these and other exquisite evolutionary adaptations contribute to the lifestyle of *N. gonorrhoeae* within human mucosal tissues.

### Human Stem Cell-Repopulated Mice

Complementing the genetics-based approaches to understand infection, recent success in the engraftment of severely immunodeficient mice with a functional human immune system (158), raises the possibility that human-restricted immune cell responses might also be explored. Highlighting the potential impact of this approach, the female genital mucosa of NOD/*LtSz-scid/scid*γ*null* (NSG) mice that have been engrafted with human CD34+ hematopoietic stem cells becomes populated with human leukocytes (159). Given that they now produce human CD4+ T cells, these mice become susceptible to chronic HIV infection. Reflecting what is observed in humans (160, 161), the lower genital tract infection with *N. gonorrhoeae* promotes localized HIV shedding without impacting systemic viral titers (159). Thus, while technically difficult and too cost-prohibitive for routine studies, this approach provides an attractive model with which to study human-specific immune responses to gonococcal infection and for translational work aiming to promote immunity or to counteract gonococcal immuno-pathogenesis.

## *N. gonorrhoeae* Manipulates the Immune Response

While early mouse infection studies aimed to understand how *N. gonorrhoeae* establishes infection, several groups have recently begun to use the model to discover how the gonococci subvert the immune response. Studies with experimentally infected female mice have shown that gonococcal infection of the genital tract leads to the production of the inflammatory Th17-associated cytokines, IL-17 and IL-22, but not specific antibodies or cytokines typical of Th1- or Th2-driven adaptive immune responses, such as IFNγ or IL-4 (137, 162). When IL-17 signaling was blocked by neutralizing antibodies or use of IL-17 receptor-deficient mice, the course of gonococcal infection was prolonged. These results indicate that innate defense mechanisms driven by IL-17, such as the recruitment of neutrophils and the secretion of anti-microbial proteins by epithelial cells, contribute to the elimination of the infection (137). Conversely, in IL-22-deficient mice, infection was more rapidly eliminated (163), implying that responses driven by IL-22 signaling promote gonococcal infection in this model. This is an unexpected result because this Th17-related cytokine tends to enhance epithelial defense by antimicrobial peptide expression and increased barrier function (164). The immune regulatory cytokine, TGFβ, was found to be important not only for the induction of the Th17 response, but also for the suppression of Th1- and Th2-driven adaptive immune responses (138, 139). Notable in this regard, administration of TGFβ-neutralizing antibodies during gonococcal infection allowed the development of anti-gonococcal antibodies and the emergence of Th1 and Th2 cells secreting IFNγ and IL-4, respectively, which correlated with the establishment of immune memory and the accelerated clearance of infection. Furthermore, re-infection of mice treated with anti-TGFβ antibody during an initial (primary) infection resulted in recall of memory responses and enhanced resistance to re-infection (138). Subsequent studies found that gonococcal infection in mice also strongly enhanced the production of another regulatory cytokine, IL-10, and induced FoxP3-negative, IL-10-dependent, type 1 regulatory T (Tr1) cells (140). Neutralizing antibodies to IL-10, or the use of IL-10-deficient mice, showed that IL-10 contributed significantly to the suppression of adaptive immunity, and that its absence allowed protective antibody responses to develop. Conventional FoxP3-positive regulatory T cells have also been observed in mice infected with *N. gonorrhoeae* (165). Combined, these findings imply that *N. gonorrhoeae* selectively induces Th17-driven innate responses that it can resist, while concomitantly suppressing Th1- and Th2-driven adaptive immune responses that would eliminate it (166). It is satisfying that humans infected with gonorrhea have also been reported to show elevated serum IL-17 (and IL-23, which enhances Th17 development) (167, 168), although these studies do not definitively demonstrate that these cytokines were elevated in response to gonococcal infection.

In considering the translational implications of the aforementioned finding that *N. gonorrhoeae* induces a TGFβ- and IL-10-dependent suppression of adaptive immune responses, it seemed reasonable that IL-12, a cytokine known for its ability to antagonize IL-10 and to drive Th1 responses, should be able to reverse this induced suppression of adaptive immunity. Accordingly, local genital administration of IL-12 encapsulated in sustained-release microparticles during gonococcal infection induced Th1-dependent immune responses with the production of IFNγ by CD4+ T cells and anti-gonococcal antibodies, and accelerated clearance of the infection. Re-infection of mice that had received IL-12 during their prior infection revealed that they had become resistant to the challenge, with the recall of anti-gonococcal antibody responses as well as IFNγ (169). An unexpected finding was that resistance to re-infection was not limited to the strain used for the initial infection, but extended to other unrelated strains, including recent clinical isolates (170). While the extent of this immune cross-protection among different gonococcal strains remains to be evaluated, it suggests that immunity is likely not dependent on strain-variable antigens such as PorB, LOS, Opa proteins, or pilin. Furthermore, immunodeficient mice lacking either B cells (and hence the ability to generate antibodies) or IFNγ did not generate resistance to re-infection in response to treatment with microencapsulated IL-12 during the primary infection (170), implying that both antibodies and IFNγ were required for immunity. While a functional role for antibodies can be readily envisaged, for example in enhancing complement-dependent bacteriolysis or phagocytic killing, or by inhibiting epithelial colonization, the contribution of IFNγ remains unclear. However, IFNγ is known to promote B cell switching to the generation of Ig isotypes that would be most efficient in these effector functions (171–173), and to upregulate the activity of phagocytes such as neutrophils and macrophages (174, 175), making these plausible determinants of protection.

While these studies provide a satisfactory explanation as to why there is no adaptive response to infection (at least within the female mouse genital tract), the underlying mechanism remains unknown. For example, it remains unclear whether the Th17-dominated response is unique to the genital tract or is instead a specific effect of gonococcal infection. If the latter, it seems reasonable to consider whether the gonococcal propensity to shed outer membrane blebs or immune-stimulatory peptidoglycan fragments (176), genomic DNA (177, 178) and/or heptose phosphates (179, 180), or to directly engage with immune-regulatory surface receptors (110, 111) may contribute to this manipulation of the immune response, but the contribution of each remains to be experimentally tested.

Through these studies, a pattern is emerging to suggest that *N. gonorrhoeae* has developed a remarkable capacity to manipulate the immune response for its own benefit, suppressing adaptive Th1- and Th2-governed responses and concomitantly eliciting Th17-driven innate responses that it is able to resist (166). The gonococcus also avoids antigen recognition by extensive variation of both the expression and specificity of several major surface antigens, and it exploits certain aspects of innate immunity, including complement C3 receptors and CEACAM molecules to attach to and invade human cells (86, 94). Moreover, it resists destruction by innate defense factors such as antimicrobial peptides and the lytic action of complement, as well as intracellular killing by neutrophils (181, 182). A corollary of these findings is that if Th1- and/or Th2-driven adaptive immune responses can be induced against constitutively expressed common antigens, then it might be possible to overcome the limitations of innate defense mechanisms by adding antigen-specific factors such as antibodies. Recent findings support these notions, and suggest that the development of an effective vaccine may indeed be feasible.

## Recent Vaccination Efforts

The induction of adaptive immune responses by administering microencapsulated IL-12 during gonococcal infection, discussed above, suggests that IL-12 functions as an adjuvant by turning the infection into a live vaccine. This finding led to the hypothesis that mice might be effectively immunized intravaginally with a vaccine consisting of gonococcal outer membrane vesicles (OMV), which retain most of the surface antigens of *N. gonorrhoeae*, plus microencapsulated IL-12 as an adjuvant (183). As predicted, this resulted in the generation of serum and genital anti-gonococcal antibodies, IFNγ production by CD4+ T cells, the establishment of immune memory that can be recalled by challenge infection 1–6 months later, and faster clearance of the challenge infection. Furthermore, the induced antibodies cross-reacted with diverse strains of *N. gonorrhoeae*, and resistance to infectious challenge was shown against heterologous as well as homologous strains (183). This study provides a clear demonstration that immunity is achievable if a suitable immunogen is appropriately administered.

The product pipeline for gonococcal vaccines is currently in the “discovery phase,” focused on the identification of vaccine targets and immune correlates of protection that they might confer (184). Progress in antigen discovery is ongoing, and has led to the identification of several conserved, stably expressed, promising vaccine targets [extensively reviewed in (181)]. Most of these antigens induce bactericidal antibodies against gonococci; some block target function, and some have shown *in vivo* efficacy in the female mouse model (32, 183, 185, 186). Candidate antigens involved in gonococcal physiology or metabolism include the transferrin receptor TbpA/B (187), which enables *N. gonorrhoeae* to access human transferrin-bound iron and is essential for urethral experimental infection of human male subjects (188), the methionine receptor MetQ (NG02139) (189, 190), nitrite reductase (AniA) (191), and phospholipase D (192). Other promising new candidates identified using a non-biased proteomics screen are involved in membrane biogenesis (BamA), LOS assembly (LptD), or translocation assembly (TamA) (190). Vaccine targets that mediate evasion of host innate defenses include MtrE, the outer membrane channel of the MtrCDE active efflux pump (193), the lysozyme inhibitor SliC (NGO1063) (190), and the Neisseria adhesion complex protein (ACP) (194). Two antigens that were identified by immunoproteomics as targets of antibodies induced by intravaginal immunization of mice with gonococcal OMV plus microencapsulated IL-12 are elongation factor (EF)-Tu and PotF3, a polyamine-binding protein (183). Both of these were also identified in proteomics profiling of cell envelopes and OMVs from *N. gonorrhoeae* strains (195). It is likely that additional targets might be identified by further immunoproteomics screening of sera and secretions taken from OMV-immunized animals.

Colonization factors are also attractive vaccine targets due to their potential to induce antibodies that block the initial establishment of infection. While pilin, the major subunit of the gonococcal pilus, is too antigenically variable to be effective, PilQ, which is the pilus secretin and thereby essential for pilus function, is an attractive target because antibodies against meningococcal PilQ are bactericidal (196). The gonococcus expresses several other outer membrane proteins that mediate adherence and/or invasion of host cells. Those for which antibodies have been shown to be bactericidal or to block interactions with host cells include OpcA (196), OmpA (197), PorB (185, 198), and the opacity (Opa) proteins (199–201). While enticing as a target because they are highly expressed on the bacterial surface and presumed to be essential for infection, as discussed above, PorB and Opa proteins are highly antigenically variable and the latter are also subject to phase variation. Moreover, while Opa expression is selected in the male urethra (96), it is selected against during menses in women (95), and by hormonally driven factors in experimentally infected female mice (125, 202). These findings illustrate that abundant and highly immunogenic surface antigens are not necessarily good vaccine targets.

As detailed above, the heptose-linked 2C7 epitope within the outer core of the gonococcal LOS is an enticing vaccine target. While it is phase-variable, there is selection for gonococci that express this epitope during human infections (31). Supporting its utility as a target, passive delivery of 2C7 monoclonal antibody is protective in mice (32). While in general polysaccharides tend to induce IgM responses with limited memory, a 2C7 peptide mimic has been developed which elicits IgG antibodies that are highly bactericidal and promote opsonophagocytic killing of gonococci, as well as *in vivo* protection against gonococcal challenge of mice (32). Interestingly, a hexamerized 2C7 monoclonal antibody that has enhanced C1q binding activity relative to the monomeric form showed increased protective activity against gonococcal infection in mice, and this was attributable to complement-mediated bactericidal activity (203).

Immune correlates and determinants of protection in the mouse model should begin to emerge as more antigens are tested for efficacy *in vivo*. Thus far, the 2C7 peptide mimic is the only vaccine for which there is strong *in vivo* evidence showing that bactericidal activity can contribute to protection against *N. gonorrhoeae* (32, 203). Antigens that showed protection against experimental murine infection in which T cell responses were examined or inferred from IgG1/IgG2a ratios include the 2C7 peptide mimic (32), gonococcal OMVs given vaginally with microencapsulated IL-12 (183), and recombinant, refolded porin (rrPorB) administered using a viral delivery system followed by rrPorB protein boosts (185). Whether serum bactericidal activity will also correlate with, or confer protection against *N. gonorrhoeae* as a result of immunization with different vaccine antigens remains to be seen. Meanwhile, broad immunologic parameters should be evaluated in future vaccine studies to help expand our understanding of these relationships.

While subunit vaccines may be effective against gonorrhea, either alone or in a “cocktail” of multiple antigens, OMV vaccines also represent an attractive approach as they contain many of the gonococcal surface antigens in their natural conformation. This approach is supported by epidemiological evidence suggesting that vaccination of humans with OMVs of the related species *N. meningitidis* might provide some protection against gonorrhea. This cross-sectional study in New Zealand examined gonorrhea rates in adolescents and adults aged 15–30 years who were given three doses of the OMV-based group B meningococcal vaccine MeNZB (204). Compared to cases of chlamydia as a control, a 31% lower rate of diagnosis with gonorrhea was found in vaccinated individuals during the follow-up period. Unfortunately, protection declined with time elapsed after immunization and was not observed in individuals who also tested positive for chlamydial infection. It is provocative that Bexsero, a recently approved serogroup B meningococcal vaccine that includes the same meningococcal OMV as used in MeNZB, does elicit antibodies that bind to *N. gonorrhoeae* (205), although serum from such individuals does not induced complement-mediated bacteriolysis of gonococci (206). Further work is required to determine whether MeNZB immunization played a causal role in the decline of gonorrhea since, for example, this could also have been attributable to continued nasal carriage of the epidemic meningococcal strain in the study population (207), or whether the apparent protection is actually attributable to cross-reactive antibody or T cell responses induced by the vaccine.

## Summary and Future Directions

Observations obtained from any experimental model, ranging from human cells or cell lines to animal infections, must be taken in context, understanding both the strengths and weaknesses of each model, and appreciating that natural infections may have different outcomes because models cannot faithfully reproduce all aspects of the relationship between a pathogen and its host. This is particularly true when their evolutionary paths overlap as much as those of *N. gonorrhoeae* and humans. Indeed, the strict specificity of *N. gonorrhoeae* for life in humans necessitates that we use every available tool to understand the molecular, metabolic, and immunologic interactions that determine the outcome of infection. As discussed throughout this review, neisserial researchers have been extraordinarily resourceful in this regard, developing infection models based upon genetically tractable cell lines; primary human epithelial cells and leukocytes; fallopian tube organ culture and cervical tissue explants; wild type, knockout and “humanized” transgenic mouse lines; and primate and human male urethral challenge models to study different aspects of infection (summarized in Table 2). When considered together, the findings from these different approaches allow a picture of the gonococcal lifestyle to begin to emerge, both remarkable in its elegance and devastating in its effectiveness as a human parasite.

**Table 2:** Pre-clinical models and pathway for gonococcal vaccine development.

**Model/species**	**Objectives/advantages**	**Remarks/limitations**
Mouse	Demonstrate contribution of non-host-restricted vaccine targets to infection or disease	Numerous defined antigens proposed based on *in vitro* studies, genome-mining, and reverse-vaccinology, but few have been evaluated so far
	Evaluate alternative or engineered immunogens	Allows prioritization of immunogens
	Evaluate alternative adjuvants	Different adjuvants may be appropriate for different immunogens
	Evaluate dose (antigen, adjuvant) and immunization routes and schedule	Will need to be re-evaluated in humans
	Determine conventional mechanisms and correlates of protection	May not fully reflect mechanisms in humans
	Identify potentially harmful antigens	Rodent toxicology may not be fully applicable to humans
Transgenic mice expressing human receptors	As above but overcomes limitations imposed by murine receptors and proteins not recognized by *N. gonorrhoeae*	Allows interaction between vaccine targets and their human receptors to be considered
Humanized immune system mice (SCID mice engrafted with human hematopoietic stem cells)	Evaluate potential responses of engrafted human immune system to *N. gonorrhoeae in vivo*	Murine genital tract imposes limits on gonococcal infection; unclear how effectively human cells are recruited to mouse tissues
Non-human primates (monkeys)	Determine cell-mediated and antibody responses	Monkeys not yet shown to be susceptible to genital gonococcal infection, thus protection not testable
	Evaluate toxicity and reactogenicity, especially in female genital tract	
*In vitro* cell and tissue culture (human)	Establish function of gonococcal virulence factors, and potential protective mechanisms based on responses identified in animals	Primary cells preferable to transformed cell lines
Humans (experimentally infected, male only)	Determine immune responses in early stages of infection	Findings may not be applicable to women
Humans (naturally infected, male and female)	Determine humoral and cellular (cytokine) responses in all stages of natural infection	Need to be re-evaluated in light of current knowledge and techniques; long-term studies not ethically permissible

Future studies must aim to further integrate these models, using each to develop new hypotheses and to validate findings observed in other systems. As this happens, each can become more sophisticated and the implications of any observations will be more effectively understood in the context of gonococcal infection and immunity. For example, ongoing efforts to “humanize” the mouse rely on cell line and primary cell-based efforts to understand gonococcal association with human cellular receptors (94), as well as blood-based studies to understand its ability to avoid serum bactericidal activity (208). Similarly, ongoing efforts to understand and then exploit gonococcal genetics have allowed *in vitro* microbiological approaches to reveal metabolic and drug resistance mechanisms that have led to studies in the established experimental human infection models (188, 209).

While not without their own pitfalls, murine infection models represent a bridge between *in vitro* cell-based studies and *in vivo* human infection. As discussed in this review, recent mouse-based studies provide a plausible explanation for why individuals with gonorrhea do not become immune to subsequent infection. This model is intellectually satisfying because it affords a hypothesis that explains how *N. gonorrhoeae* can repeatedly infect individuals and thereby persist in the population, and it also provides insight as to what types of immune response might be effective in combatting the pathogen. Future mouse-based studies should reveal whether this outcome reflects the normal response of the female genital tract to bacteria, or whether it represents instead a unique characteristic of *N. gonorrhoeae*, providing fundamental insight regarding infection, immunity, and immuno-pathogenesis. Taken together, the findings obtained from all experimental systems, including *in vitro* cell- and tissue-based systems, animal models, and human studies afford a novel paradigm within which to formulate and refine new hypotheses for testing, thereby providing new insights into this complex and vexing infection.

The ultimate goal of these efforts is to develop new interventions to stop the spread of *N. gonorrhoeae*, an aim that is particularly enticing considering that the restricted host niche might make it possible for this pathogen to be eradicated once a vaccine is in hand (210, 211). While we have passed through a period in which gonorrhea was often considered as a low priority afterthought, the recent rapid increase in rates of infection and the emergence of multidrug-resistant “superbug” strains of *N. gonorrhoeae* has re-awoken the public's appreciation of this emerging threat, and re-invigorated public health and industrial interest in developing a gonococcal vaccine. This attention has come at an opportune time, since recent work has offered new insight into what types of response can confer protection, revealed new targets for vaccine development, and provided ever-more sophisticated models in which to test their efficacy. Hopefully, this momentum will rapidly bring us to a gonococcal vaccine.

## Author's Note

The contents of this publication are the sole responsibility of the authors and do not necessarily reflect the views, opinions, or policies of Uniformed Services University, the Henry M. Jackson Foundation for the Advancement of Military Medicine or the Department of Defense.

## Author Contributions

All authors contributed equally to the conception and writing of this review, and approved the final version.

### Conflict of Interest

MR serves as a paid consultant for TherapyX, Inc., which is developing sustained release microparticulate adjuvants for use in inflammatory disease therapy and gonococcal vaccine development. SG-O is co-founder of Engineered Antigens Inc., which is focused on protein structure-based design of vaccine immunogens targeting pathogens including *N. gonorrhoeae*. The remaining author declares that the research was conducted in the absence of any commercial or financial relationships that could be construed as a potential conflict of interest.
